# Ultrasonographic Median Nerve Cross-Sectional Area and Clinical, Electrodiagnostic, and Laboratory Biomarkers in Electrodiagnostically Confirmed Carpal Tunnel Syndrome: A Single-Center Correlational Study

**DOI:** 10.3390/diagnostics15182407

**Published:** 2025-09-22

**Authors:** Hasan Kara, Hüseyin Kaplan, Fatma Nur Aba, Servin Karaca, İsa Cüce

**Affiliations:** 1 Department of Physical Medicine and Rehabilitation, Erciyes University Faculty of Medicine, 38039 Kayseri, Turkey; sarvinmoloudnejad72@gmail.com (S.K.); dr.icuce@hotmail.com (İ.C.); 2 Department of Rheumatology, Aksaray Training and Research Hospital, 68200 Aksaray, Turkey; hkaplan_87@hotmail.com; 3 Department of Physical Medicine and Rehabilitation, Nevşehir State Hospital, 50300 Nevşehir, Turkey; fatmanuraslan05@gmail.com

**Keywords:** cross-sectional area, blood tests, signs and symptoms, median nerve, carpal tunnel syndrome, ultrasonography, electrodiagnosis, nerve conduction studies

## Abstract

**Objectives:** This study aimed to evaluate the relationship between the median nerve cross-sectional area (CSA, mm^2^) and clinical findings, blood test results, and electrodiagnostic (EDX) measurements in patients with carpal tunnel syndrome (CTS). **Methods:** This cross-sectional study included 62 patients (111 hands). The median nerve CSA was assessed using ultrasound (US). The clinical assessment included symptom duration, symptom severity, the Boston Carpal Tunnel Questionnaire (BCTQ), and physical examination. Patient-level analyses used the CSA of the most symptomatic hand for clinical and laboratory variables (*n* = 62 patients). Hand-level EDX analyses accounted for within-patient clustering by reporting right and left hands separately. Associations were summarized with Spearman’s ρ and 95% confidence intervals (CIs); multiplicity was addressed using Benjamini–Hochberg false discovery rate (FDR). EDX units: latency ms, amplitude mV/µV, and velocity m/s. **Results:** CSA was not associated with global symptom burden (Visual Analog Scale; BCTQ). No laboratory marker remained significant after FDR across the full panel. By contrast, CSA correlated with EDX impairment at the hand level with low-to-moderate effect sizes; for example, distal motor latency was positively associated with CSA on the right (ρ = 0.557, 95% CI 0.334–0.733) and left (ρ = 0.318, 95% CI 0.022–0.578). CSA also correlated positively with CTS EDX severity (right: ρ = 0.449, 95% CI 0.223–0.646; left: ρ = 0.354, 95% CI 0.071–0.609). **Conclusions:** Ultrasonographic CSA was associated with electrophysiologic impairment and was not associated with overall symptom burden; laboratory signals did not survive FDR control. Accordingly, CSA may serve as a complementary morphologic adjunct to clinical assessment and EDX, with limited utility as a stand-alone severity metric.

## 1. Introduction

Carpal tunnel syndrome (CTS) is a common peripheral neuropathy caused by median nerve compression at the wrist, typically presenting with pain, numbness, tingling, and weakness in the thumb, index, middle, and part of the ring finger [1,2,3]. CTS imposes significant restrictions on both the quality of life and work efficiency, necessitating accurate diagnosis, timely treatment, and effective management [4,5]. The conventional approach for diagnosing CTS involves a combination of clinical assessment, physical examination, and electrodiagnostic (EDX) studies [6]. Nerve conduction studies (NCSs) are widely used as the clinical reference standard [6,7]. While highly specific, EDX has practical limitations (e.g., occasional false negatives, patient discomfort, and access constraints), encouraging a shift toward complementary modalities [8,9].

Musculoskeletal ultrasound (US) has become an increasingly used, non-invasive technique for evaluating median nerve pathology in CTS in recent times. A common ultrasonographic measure is the median nerve’s cross-sectional area (CSA) at the pisiform bone level [10]. An increased CSA is associated with nerve swelling, a hallmark of CTS. US has several strengths: it is noninvasive, accessible, cost-effective, and allows dynamic visualization of the carpal tunnel, adjacent joints, and tendons. A meta-analysis reported that ultrasonography had a pooled sensitivity of 77.6% (95% CI: 71.6–83.6%) and specificity of 86.8% (95% CI: 78.9–94.8%) in diagnosing CTS [11]. However, the correlation between CSA and symptoms remains disputed, with conflicting evidence. CSA also correlates with anthropometry (e.g., body weight and BMI) in asymptomatic individuals, and measurement heterogeneity (landmark, tracing method, and thresholds), operator dependence, and comorbidities (e.g., diabetes and thyroid dysfunction) may contribute to inconsistent findings [12,13,14]. Thus, although the median nerve CSA demonstrates some association with physical and clinical parameters, its role in reflecting disease severity and progression in CTS is complex and unreliable.

Median nerve CSA was significantly correlated with various EDX findings in patients with CTS. An increased median CSA correlates with a delayed onset latency of the compound motor action potential (CMAP) and decreased CMAP amplitude in non-diabetic individuals with CTS [15]. This suggests that motor conduction becomes more impaired as the median nerve swells. The CSA measured at the carpal tunnel inlet positively correlates with the neurophysiological severity of CTS, as determined by NCS [16]. Although many imaging studies (US and MRI) have evaluated the diagnostic and severity-related utility of median nerve CSA in CTS, evidence linking CSA to routine blood biomarkers is sparse and largely confined to condition-specific cohorts (e.g., thyroid dysfunction), with small samples and heterogeneous methods; consistent associations have not been established [17,18,19,20,21]. The current literature predominantly emphasizes EDX tests and imaging techniques, with minimal attention paid to blood-based biomarkers. Unlike prior studies, this study evaluated routine blood tests (e.g., thyroid parameters) alongside a standardized ultrasound protocol in a single-center cohort of EDX-confirmed CTS and systematically examined their associations with CSA within the same sample. Evaluating correlations with routine laboratory biomarkers may be important because it may capture systemic endocrine–metabolic influences on median nerve morphology (e.g., edema, extracellular matrix, and microvascular changes) that nerve-centric measures might miss.

Therefore, rather than testing diagnostic accuracy, this study aimed to quantify associations between US-derived median nerve CSA and (1) EDX measures, (2) clinical findings, and (3) routine laboratory parameters in patients with CTS.

## 2. Materials and Methods

### 2.1. Study Design and Participants

This study was conducted at the Department of Physical Medicine and Rehabilitation (PMR) of the Erciyes University Faculty of Medicine between 1 January 2024 and 1 February 2025. Patients referred to the electroneuromyography (ENMG) unit with a preliminary diagnosis of CTS following routine clinical evaluation and subsequently diagnosed with CTS based on electrodiagnostic tests were considered for inclusion. Patients were consecutively recruited from those referred to the ENMG unit, and no randomization was applied. Referral sources included physical medicine & rehabilitation (PMR), neurology, neurosurgery, orthopedics and hand surgery clinics. Only electrodiagnostically positive CTS cases were included in the study. Those with clinical CTS but normal NCS were excluded regardless of ultrasound findings. Bilateral symptoms were common (53/62; 85.5%).

The inclusion criteria were as follows: having clinical symptoms consistent with CTS, being between 18 and 70 years of age, and confirmation of the CTS diagnosis through EDX studies. All participants were required to have adequate cognitive and physical abilities to complete the clinical assessments and questionnaires.

The exclusion criteria included a diagnosis of diabetes mellitus; the presence of polyneuropathy; a history of rheumatologic disease; previous surgery or fracture in the affected upper extremity; systemic neurological conditions such as neuromuscular junction disorders or motor neuron disease; known thyroid disease; pregnancy; the presence of a bifid median nerve on ultrasonographic examination; space-occupying lesions within the carpal tunnel; peripheral nerve disorders unrelated to CTS; renal insufficiency; and chronic neck pain lasting at least three months and/or clinical findings indicative of cervical radiculopathy.

The exclusion criteria were assessed based on patients’ medical history and electronic health records. Routine laboratory screening, including thyroid function tests, was not systematically performed for all participants unless clinically indicated.

Written informed consent was obtained from all participants prior to enrollment. The study was approved by the local ethics committee (No. 2023/827) and adhered to the principles outlined in the Helsinki Declaration.

Patients meeting the inclusion criteria were evaluated for demographic characteristics, including age, sex, height, weight, BMI, marital status, occupation, educational level, and dominant hand. Detailed medical histories were obtained, including current medications, history of surgeries, known comorbidities, and lifestyle factors, such as smoking and alcohol use.

### 2.2. Clinical Evaluation

Clinical evaluation followed a standardized protocol. Typical symptoms were recorded using a structured checklist (nocturnal paresthesia; numbness/tingling in the thumb, index, middle, and radial half of the ring finger; and activity-related exacerbation). Provocative maneuvers, such as Phalen’s and Tinel’s at the carpal tunnel, and carpal compression, were considered positive if they reproduced paresthesia in the median distribution. Examination included sensory mapping (light touch and pinprick) across median, ulnar, and radial territories and motor assessment of abductor pollicis brevis (APB) strength with inspection for thenar atrophy. Only patients with clinical findings of CTS and EDX confirmation (per predefined NCS criteria) were included.

The severity of CTS-related symptoms was evaluated using the Visual Analog Scale (VAS). Patient-level analyses used the clinically most symptomatic hand, defined as the side with the higher VAS pain score. In bilateral cases with equal VAS scores, the hand with greater electrodiagnostic severity was selected. All patients completed the Boston Carpal Tunnel Questionnaire (BCTQ). The BCTQ is used to assess the severity of symptoms and the functional status of patients with carpal tunnel syndrome. This process consists of two parts [22].

1. Symptom Severity Scale (SSS): This section assesses the intensity and frequency of symptoms, including pain, numbness, and tingling in the hand and wrist.
2. Functional Status Scale (FSS): This section assesses how carpal tunnel syndrome affects a person’s ability to perform daily activities, such as writing, buttoning clothes, or holding objects.

### 2.3. Ultrasonographic Assessment

Ultrasonographic assessments were performed in the PMR department’s musculoskeletal US unit by a specialist with ten years of experience in musculoskeletal sonography. All sonographic measurements were performed using a GE Logiq P8 US system (Application software version R4, Software revision 5.2; manufacturer: GE HealthCare Technologies, Inc., Chicago, IL, USA), and US examinations were performed using a high-frequency linear transducer (3–12 MHz).

**Figure 1:**
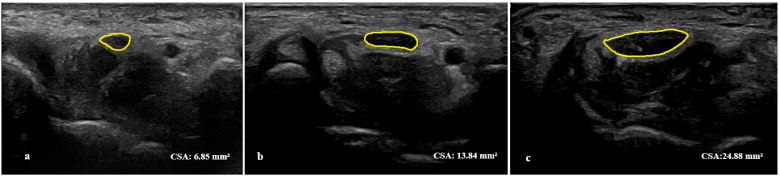
Ultrasonographic measurement of median nerve CSA at the carpal tunnel inlet. The yellow line indicates the traced border of the median nerve CSA. (**a**) CTS with normal-range CSA (CSA = 6.85 mm^2^). 36-year-old male, left-hand dominant; symptoms 4 months; BCTQ SSS 1.45, FSS 1.38; VAS 5/10. EDX severity mild, key NCS values: DML 3.43 ms, digit-to-wrist sensory latency 3.63 ms, CMAP amplitude 17.2 mV, SNAP amplitude 22.7 µV, sensory velocity 31.7 m/s. (**b**) CTS with increased CSA (CSA = 13.84 mm^2^). 65-year-old female, left-hand dominant; symptoms 24 months; BCTQ SSS 2.91, FSS 2.63; VAS 8/10. EDX severity moderate, key NCS values: DML 5.98 ms, digit-to-wrist sensory latency 5.4 ms, CMAP amplitude 6.2 mV, SNAP amplitude 5 µV, sensory velocity 22.2 m/s. (**c**) CTS with markedly increased CSA (CSA = 24.88 mm^2^), consistent with severe disease. 67-year-old female; right-hand dominant; symptom duration 60 months; BCTQ SSS 3.55, FSS 3.88; VAS 10/10. EDX severity: severe; CMAP and SNAP were unobtainable/absent on nerve conduction studies (DML not measurable).

Participants were examined with the forearm supinated and the wrist in neutral position, using generous gel and minimal transducer pressure. Median nerve CSA was measured bilaterally in the transverse plane at the carpal tunnel inlet (pisiform level, proximal to the flexor retinaculum). Imaging depth was set to approximately (2.5–3.5) cm with the focal zone placed at the level of the nerve. The nerve was identified by its fascicular pattern and a hyperechoic epineurial rim; CSA was obtained by continuous tracing along the inner border of the epineurium, following published recommendations (Figure 1) [23,24]. Three short-axis acquisitions were obtained with probe lift and repositioning. To standardize edge definition and minimize compression artefacts, the highest-quality frame was selected, and CSA was traced three times on this single frozen image; the mean of the three tracings (mm^2^) was used for analysis.

Intra-rater reliability was assessed by repeated CSA measurements in 20 randomly selected cases, and the intraclass correlation coefficient (ICC) indicated excellent agreement (ICC = 0.998, 95% CI: 0.995–0.999, *p* < 0.001).

### 2.4. Electrodiagnostic Studies

Standardized nerve conduction studies were conducted for all participants using a Natus ENMG system (Natus Dantec Keypoint, Software version 2.40; manufacturer: Natus Medical Inc., Middleton, WI, USA) in a temperature-controlled room (maintained at 24–26 °C) to avoid variability in the conduction velocities. All procedures were performed by an experienced physician certified in clinical neurophysiology. Electrodiagnostic evaluation included both motor and sensory nerve conduction studies of the median nerve. For the sensory conduction study, orthodromic sensory nerve action potentials (SNAPs) were recorded by stimulating the second digit and measuring them at the wrist. Peak latency (in milliseconds), amplitude (in microvolts), and conduction velocity (in m/s) were measured. Motor conduction was assessed by stimulating the median nerve at both the wrist and elbow, with CMAPs and F-wave latency recorded from the abductor pollicis brevis muscle. Measurements included distal motor latency (DML) in milliseconds and CMAP amplitude in millivolts.

Ulnar nerve studies were also performed to exclude other causes of neuropathy and for differential diagnoses. Hand skin temperature was kept above 32 °C prior to conducting the tests.

EDX confirmation of CTS was established in the clinically affected limb when median sensory conduction across the wrist was abnormal—i.e., prolonged peak latency and/or reduced conduction velocity over the digit-to-wrist segment with normal ulnar responses recorded under identical conditions—and/or when the median distal motor latency (DML) to the abductor pollicis brevis over an 8 cm segment exceeded our laboratory upper limit of normal [25]. CTS severity was graded according to our laboratory reference limits. Categories were defined as follows:

- Negative: All standard median studies within normal limits.
- Mild: Abnormal sensory conduction across the wrist (prolonged sensory latency/decreased velocity), DML within normal limits.
- Moderate: Abnormal sensory conduction and prolonged DML.
- Severe: Absent SNAP, low-amplitude or absent CMAP (DML prolonged).

### 2.5. Laboratory Assessments

Blood test data collected within the previous three months were obtained from the hospital’s electronic health record system. The evaluated laboratory parameters were grouped into the following categories:

Vitamin and Hormonal Profiles: This included 25-hydroxyvitamin D, vitamin B12, thyroid-stimulating hormone (TSH), free triiodothyronine (fT3), and free thyroxine (fT4).

Inflammatory and Metabolic Markers: Erythrocyte sedimentation rate (ESR), C-reactive protein (CRP), blood urea nitrogen (BUN), serum creatinine, estimated glomerular filtration rate (eGFR), and uric acid levels were recorded.

Liver Enzymes: Aspartate aminotransferase (AST) and alanine aminotransferase (ALT) were included to assess liver function.

Complete Blood Count (CBC) and Derived Inflammatory Ratios: Hemoglobin (Hb), hematocrit (HCT), white blood cell (WBC) count, platelet count (PLT), red blood cell (RBC) count, mean corpuscular volume (MCV), mean corpuscular hemoglobin (MCH), mean corpuscular hemoglobin concentration (MCHC), red cell distribution width (RDW-CV and RDW-SD), and immature granulocyte (IG) percentage, neutrophil-to-monocyte ratio (NMR), lymphocyte-to-monocyte ratio (LMR), platelet-to-lymphocyte ratio (PLR), and neutrophil-to-lymphocyte ratio (NLR).

### 2.6. Statistical Analysis

All analyses were performed in IBM SPSS Statistics for Windows, version 22.0 (Armonk, NY, USA). Distributional assumptions were checked with Shapiro–Wilk tests and Q–Q plots. Associations between ultrasonographic median nerve CSA and clinical, laboratory, or EDX variables were examined primarily with Spearman’s rank correlation (ρ); Pearson’s r was used only when both variables were approximately normally distributed. For patient-level analyses (clinical and laboratory variables), the unit of analysis was the patient and CSA from the most symptomatic hand was used. EDX analyses were hand-level and are reported separately for right and left hands. To control for multiplicity, the Benjamini–Hochberg false discovery rate (FDR) was applied within each side-specific EDX family and across the entire laboratory panel; FDR-adjusted q-values are reported alongside raw *p*-values. Statistical significance was set at *p* < 0.05. Correlation coefficients were interpreted as follows: 0.00–0.30 (or 0.00 to −0.30) = negligible correlation, 0.30–0.50 (or −0.30 to −0.50) = low positive (negative) correlation, 0.50–0.70 (or −0.50 to −0.70) = moderate positive (negative) correlation, 0.70–0.90 (or −0.70 to −0.90) = high positive (negative) correlation, and 0.90–1.00 (or −0.90 to −1.00) = very high positive (negative) correlation [26].

## 3. Results

### 3.1. Demographic and Clinical Characteristics

**Figure 2:**
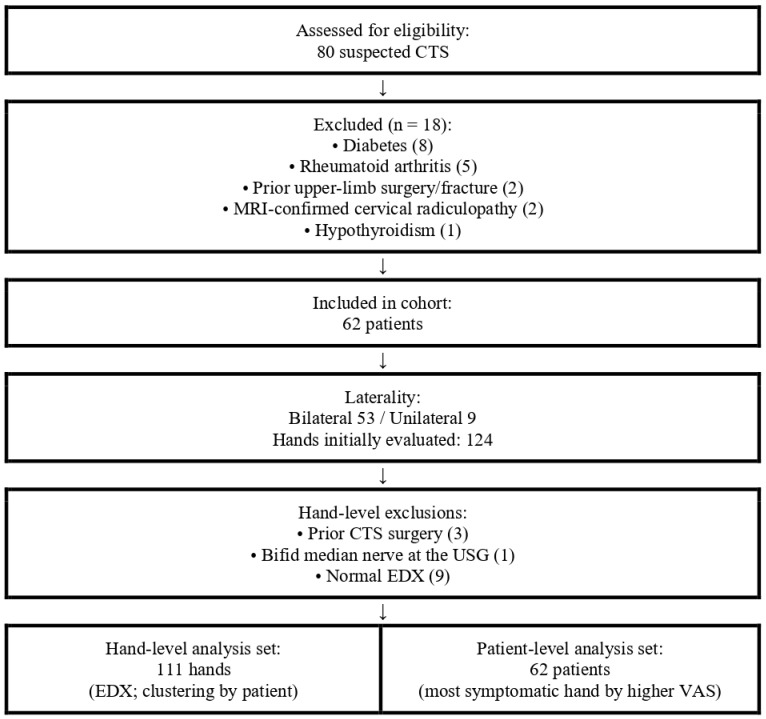
Flow of Participants and Analysis Sets.

A total of 80 suspected CTS cases were assessed; 18 were excluded (diabetes 8, rheumatoid arthritis 5, prior surgery/fracture 2, MRI-confirmed radiculopathy 2, and hypothyroidism 1), yielding 62 patients (Figure 2). Bilateral symptoms were frequent (53/62; 85.5%), producing 124 hands initially; after excluding 3 hands with prior CTS surgery, 1 bifid median nerve, and 9 with normal EDX, 111 hands entered hand-level analyses (Figure 2). Patient-level clinical/lab analyses used the most symptomatic hand (higher VAS). EDX analyses were performed at the hand level and are reported separately for right and left hands. Participant characteristics: age 51.56 ± 9.34 years, 72.6% female, BMI 31.02 ± 5.26 kg/m^2^, symptom duration 19.9 ± 15.9 months; hypertension (24.2%) and hyperlipidemia (8.1%) were the most common comorbidities, while diabetes and overt thyroid disease were excluded by design (Table 1). EDX severity (hand-level, *n* = 111) was mild 52.3%, moderate 40.5%, and severe 7.2% (Table 2).

### 3.2. Correlation Between Median Nerve CSA and Clinical Parameters

Analyses were conducted using the most symptomatic hand of each patient. No statistically significant correlations were observed between the symptomatic-side median nerve CSA and VAS, BCTQ total and subscales (SSS and FSS), symptom duration, or anthropometric variables such as age, height, weight, or BMI (*p* > 0.05).

Among BCTQ sub-items, CSA showed a negligible but statistically significant positive correlation with “How often did hand numbness or tingling wake you up during a typical night during the past two weeks?” (*n* = 62, ρ = 0.281, 95% CI: 0.056–0.489, *p* = 0.027, q = 0.19). CSA also demonstrated a low positive correlation with “How severe is numbness (loss of sensation) or tingling at night?” (*n* = 62, ρ = 0.312, 95% CI: 0.059–0.543, *p* = 0.014, q = 0.15), which assesses nocturnal paresthesia. Additionally, CSA demonstrated a low positive correlation with the item “Buttoning of clothes” (*n* = 62, ρ = 0.329, 95% CI: 0.068–0.545, *p* = 0.009, q = 0.15), which evaluates the ability to fasten buttons. However, after Benjamini–Hochberg FDR correction within the BCTQ item family, none remained significant (all q > 0.05).

### 3.3. Correlation Between Median Nerve CSA and Laboratory Parameters

Using the CSA of the most symptomatic hand, no laboratory marker remained significant after Benjamini–Hochberg FDR control across the full panel. Nominally, serum fT4 showed a moderate negative association with CSA (*n* = 27, ρ = −0.576, 95% CI −0.752 to −0.298, *p* = 0.005; q = 0.18) and RDW-CV a negligible negative association (*n* = 58, ρ = −0.272, 95% CI −0.523 to −0.011, *p* = 0.040; q = 0.51), but both signals did not survive multiplicity adjustment.

No statistically significant correlations were found between the symptomatic median nerve CSA and other laboratory parameters examined, including vitamin D, CRP, BUN, creatinine, GFR, uric acid, AST, ALT, complete blood count indices (WBC, Hb, PLT, neutrophils, lymphocytes, monocytes, eosinophils, basophils, RBC, HCT, MCV, MCH, MCHC, and RDW-SD), other hematologic ratios (LMR, NMR, PLR, and NLR), ESR, vitamin B12, TSH, and fT3 (all *p* and q > 0.05).

### 3.4. Correlation Between Median Nerve CSA and EDX Findings

Correlation analysis was conducted between the median nerve CSA and EDX parameters for both hands. On the right side, CSA was moderately positively correlated with DML, lowly negatively correlated with CMAP amplitude, and negligibly positively correlation with F-wave latency. No significant correlation was observed with motor conduction velocity (*p* and q > 0.05). In sensory conduction studies, CSA showed a low positive correlation with digit-to-wrist latency and a low negative correlation with SNAP amplitude and conduction velocity (Table 3 and Table 4).

Similarly, on the left side, the CSA demonstrated a low positive correlation with DML and F-wave latency. Conversely, a low negative correlation was observed with CMAP amplitude. Among the sensory parameters, CSA showed a low negative correlation with SNAP amplitude and a low positive correlation with sensory latency. Motor conduction velocity (wrist–elbow) and sensory velocity measurements on the left side did not show any statistically significant correlations (*p* and q > 0.05) (Table 3, Table 4 and Table 5).

A statistically significant low positive correlation was observed between median nerve CSA and CTS severity (electrodiagnostic grading) in both hands (right: ρ = 0.449, 95% CI: 0.223–0.646, *p* < 0.001, q < 0.001) (left: ρ = 0.354, 95% CI: 0.071–0.609, *p* = 0.012, q = 0.012).

## 4. Discussion

In this single-center cohort of EMG-confirmed CTS, our most robust findings—reported at the hand level and after FDR control—were the electrodiagnostic associations of ultrasonographic median nerve CSA. Larger CSA correlated moderately with DML and weakly with F-wave and digit-to-wrist sensory latency, while low negative correlations were observed with CMAP and sensory amplitudes and with sensory conduction velocity. By contrast, CSA showed no association with global symptom burden (VAS and BCTQ totals), and no laboratory biomarker remained significant after FDR.

Previous studies have reported inconsistent associations between CSA and clinical severity, with some showing moderate correlations with symptom scores, while others found weak or no significant relationships [27,28,29,30,31]. This variability likely reflects differences in study design, patient mix, and ultrasound methodology [32,33]. In our study, CSA was not associated with global symptom burden—VAS and BCTQ total (SSS/FSS)—or symptom duration, and this remained true after FDR control. Item-level signals for nocturnal paresthesia and fine motor tasks were negligible and did not persist after FDR, indicating that CSA does not capture overall symptom burden. Notably, meta-analytic data indicate that ultrasonography can discriminate CTS from non-CTS with moderate accuracy (pooled sensitivity ≈ 77.6% and specificity ≈ 86.8%) [11]; however, such between-group diagnostic performance does not imply a strong within-cohort correlation between CSA and symptom severity. Accordingly, CSA should be interpreted as an adjunctive measure alongside clinical and electrodiagnostic findings rather than a standalone severity index for clinical decision-making.

In our study, ultrasonographic median nerve CSA showed statistically significant low-to-moderate correlations with several electrodiagnostic parameters after Benjamini–Hochberg FDR control. Specifically, larger CSA values were associated with prolonged distal motor and sensory latencies and F-latency as well as reduced CMAP and SNAP amplitudes. These findings align with the known pathophysiology of CTS, wherein compression of the median nerve results in structural changes (e.g., nerve swelling as reflected by increased CSA) and functional impairments (e.g., slowed conduction and decreased amplitudes). Previous studies have similarly reported moderate to strong associations between CSA and distal latency. However, the relationship between CSA and amplitude measures tends to be weaker and more variable, likely due to influences such as axonal degeneration or technical variability [15,21,34]. Martikkala et al. [16] reported a positive association between wrist CSA and neurophysiological CTS severity (r = 0.619, *p* < 0.001), Mohammadi et al. [35] likewise found a significant CSA–severity correlation (*p* < 0.008), whereas Kang et al. [28] noted that although wrist CSA differed between controls and CTS, it failed to distinguish mild from moderate CTS—unlike the wrist-to-forearm ratio. In our study, we observed low positive correlations between CSA and EDX severity on both sides, which remained significant after FDR. These findings are consistent with previous studies reporting that increased disease severity is accompanied by greater nerve enlargement. However, the modest strength of these correlations suggests that while CSA can provide structural information about nerve swelling, it should not be used in isolation to assess disease severity.

At the patient level (most symptomatic hand), no laboratory marker remained significant after Benjamini–Hochberg FDR control across the full panel. Nominal signals were observed for a moderate negative correlation between CSA and fT4 and a negligible negative correlation with RDW-CV, but neither survived multiplicity adjustment, and we therefore interpret them as hypothesis-generating rather than confirmatory. While thyroid dysfunction is an established risk factor for CTS [36,37,38], prior reports have chiefly linked specific blood tests to electrodiagnostic abnormalities, and associations with CSA have been inconsistent [39,40,41]. Larger, multicenter, longitudinal studies will be required to confirm or refute these signals and to clarify their clinical relevance.

From a measurement standpoint, ultrasonographic CSA offers a practical, standardized, and reproducible morphologic readout in specialist practice. In this study, we used a predefined protocol (transverse acquisition at the carpal tunnel inlet/pisiform, continuous tracing along the inner epineurial border, and minimal transducer pressure) and obtained excellent ICC, underscoring feasibility in routine practice. After FDR control, CSA tracked neurophysiological impairment with low-to-moderate effect sizes, indicating incremental value as a structural complement rather than a replacement for nerve conduction studies. In this context, when nerve conduction studies are unavailable or inconclusive, ultrasonographic CSA may serve as a complementary diagnostic tool. In a specialist workflow, ultrasound can add value by (i) confirming and localizing morphologic changes, (ii) detecting structural variants or coexisting pathology (e.g., bifid median nerve, space-occupying lesion, and flexor tenosynovitis), and (iii) guiding injection procedures when indicated, while also providing a quick, low-burden point-of-care assessment. By contrast, MRI can depict carpal tunnel anatomy in great detail but is less feasible for routine CTS work-ups due to cost and access constraints [20]. Accordingly, we favored ultrasonographic CSA measurement, a non-invasive, cost-effective, and widely accessible modality for routine practice. Overall, CSA should be integrated with clinical examination and EDX as a complementary measurement rather than used to grade symptom severity on its own.

This study has limitations. First, its cross-sectional, single-center design and modest sample limit causal inference and generalizability. Second, the absence of a control group of healthy individuals prevents the determination of specific cutoff values for CSA and limits the assessment of diagnostic specificity. Another limitation of our study is that patients with normal electrodiagnostic findings but increased CSA were not included. Therefore, the diagnostic value of ultrasound in electrodiagnostically negative CTS cases could not be addressed. Another limitation of this study is that laboratory values were collected within a three-month window prior to ultrasonographic and electrodiagnostic assessments, which may introduce temporal variability. Future studies should use synchronous data collection to ensure greater consistency.

## 5. Conclusions

Ultrasonographic median nerve CSA correlated with electrodiagnostic impairment but not with overall symptom burden; no laboratory marker remained significant after FDR control. CSA provides morphologic context that complements clinical examination and nerve conduction studies, but it should not be used alone to grade clinical severity. Ultrasound may add value when NCSs are unavailable or inconclusive and for detecting structural variants. Although ultrasound-based CSA has been reported to show acceptable diagnostic sensitivity and specificity, this does not imply a strong within-cohort correlation with clinical severity. Multicenter, longitudinal studies using standardized ultrasound protocols are needed to establish a clinically meaningful CSA threshold.

## Figures and Tables

**Table 1:** Demographic, clinical, and ultrasonographic characteristics of participants.

Variable	Overall (*N*: 62 Patients)
Age, years	51.56 ± 9.34
Gender, Female/Male, *n* (%)	45/17 (72.6/27.4)
BMI, kg/m^2^	31.02 ± 5.26
Dominant hand, Right/Left, *n* (%)	58/4 (93.5/6.5)
Educational Status, *n* (%)	
Illiterate	8 (12.9)
Primary or Secondary School:	34 (54.8)
High School	15 (24.2)
University	5 (8.1)
Occupational Status, *n* (%)	
Housewife	38 (61.3)
Office workers	1 (1.6)
Worker/Laborer	4 (6.5)
Retired	5 (8.1)
Self-employed	9 (14.4)
Other	5 (8.1)
Comorbidity, *n* (%)	
None	33 (53.2)
Hypertension	15 (24.2)
Hyperlipidemia	5 (8.1)
Pulmonary disease	4 (6.5)
Cardiovascular disease	3 (4.8)
Osteoporosis	2 (3.2)
Symptom duration, months	19.9 ± 15.9
Laterality, Bilateral/Unilateral, *n* (%)	53/9 (85.5/14.5)
VAS pain (0–10)	6.85 ± 2.46
BCTQ-SSS (1–5)	2.58 ± 0.72
BCTQ-FSS (1–5)	2.57 ± 0.90
Median nerve CSA, mm^2^—right	13.42 ± 4.26 (range: 6.73–26.2)
Median nerve CSA, mm^2^—left	13.42 ± 3.84 (range: 7.88–24.4)
Median nerve CSA, mm^2^—most symptomatic side	14.24 ± 4.34 (range: 7.43–26.2)

Continuous variables are presented as mean ± SD; categorical variables as *n* (%). VAS, Visual Analog Scale (0–10). BCTQ-SSS, Boston Carpal Tunnel Questionnaire—Symptom Severity Scale (1–5). BCTQ-FSS, Function Severity Scale (1–5). CSA, Cross-Sectional Area. BMI, Body Mass Index.

**Table 2:** Electrodiagnostic severity distribution.

Severity Category	Hands, *n* (%)
Mild	58 (52.3)
Moderate	45 (40.5)
Severe	8 (7.2)
Total	111 (100)

Counts reflect hands with complete electrodiagnostic data (*n* = 111).

**Table 3:** FDR-adjusted significant correlations between ultrasonographic median nerve CSA and EDX parameters.

Side	Variable	ρ (Spearman)	95% CI	*n*	*p*	q (FDR)
EDX-Right	Motor wrist latency	0.557	0.334–0.733	58	<0.001	<0.001
EDX-Right	Sensory latency	0.486	0.222–0.688	57	<0.001	0.003
EDX-Right	Motor F-latency	0.288	0.003–0.544	56	0.033	0.039
EDX-Right	Sensory amplitude	−0.411	−0.639 to −0.144	60	0.002	0.004
EDX-Right	Sensory velocity	−0.419	−0.628 to −0.157	57	0.001	0.002
EDX-Right	Motor wrist amplitude	−0.330	−0.548 to −0.057	60	0.014	0.020
EDX-Left	Motor wrist latency	0.318	0.022–0.578	49	0.030	0.042
EDX-Left	Motor F-latency	0.321	0.043–0.570	49	0.028	0.042
EDX-Left	Motor wrist amplitude	−0.327	−0.572 to −0.049	50	0.025	0.042
EDX-Left	Sensory amplitude	−0.363	−0.596 to −0.068	50	0.012	0.042
EDX-Left	Sensory latency	0.319	0.051–0.581	47	0.029	0.042

ρ: Spearman correlation coefficient; CI: confidence interval; q: Benjamini–Hochberg false discovery rate (FDR)–adjusted *p* within side-specific EDX families.

**Table 4:** Correlation Matrix Between Ultrasonographic Right Median Nerve CSA and EDX Parameters.

	Cross-Sectional Area	Motor Wrist Latency	Motor Wrist Amplitude	Motor Wrist–Elbow Velocity	Motor F-Latency	Sensory Latency	Sensory Amplitude	Sensory Velocity
Cross-Sectional Area	1	0.557	−0.33	0.021	0.288	0.486	−0.411	−0.419
Motor Wrist Latency	<0.001	1	−0.376	−0.101	0.528	0.706	−0.496	−0.72
Motor Wrist Amplitude	0.014	0.005	1	0.166	−0.258	−0.34	0.371	0.328
Motor Wrist–Elbow Velocity	0.881	0.463	0.227	1	−0.194	−0.188	−0.102	0.101
Motor F-Latency	0.033	<0.001	0.058	0.155	1	0.59	−0.505	−0.426
Sensory Latency	<0.001	<0.001	0.011	0.17	<0.001	1	−0.433	−0.892
Sensory Amplitude	0.002	<0.001	0.005	0.457	<0.001	0.001	1	0.378
Sensory Velocity	0.001	<0.001	0.015	0.461	0.001	<0.001	0.004	1
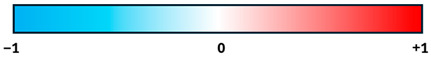								

Cells in the upper triangle display Spearman’s ρ; cells in the lower triangle show two-sided *p*-values. Color legend: red = positive, blue = negative; saturation increases with |ρ| (white ≈ 0). The color scale is fixed from −1 (dark blue) to +1 (dark red) across all correlation tables; diagonal = 1.0 (self-correlation).

**Table 5:** Correlation Matrix Between Ultrasonographic Left Median Nerve CSA and EDX Parameters.

	Cross-Sectional Area	Motor Wrist Latency	Motor Wrist Amplitude	Motor Wrist–Elbow Velocity	Motor F-Latency	Sensory Latency	Sensory Amplitude	Sensory Velocity
Cross-Sectional Area	1	0.318	−0.327	−0.276	0.321	0.319	−0.363	−0.227
Motor Wrist Latency	0.03	1	−0.401	−0.093	0.67	0.805	−0.683	−0.766
Motor Wrist Amplitude	0.025	0.005	1	0.352	−0.366	−0.21	0.329	0.265
Motor Wrist–Elbow Velocity	0.06	0.536	0.015	1	−0.334	−0.101	0.77	0.26
Motor F-Latency	0.028	<0.001	0.011	0.22	1	0.58	−0.533	−0.393
Sensory Latency	0.029	<0.001	0.157	0.497	<0.001	1	−0.675	−0.879
Sensory Amplitude	0.012	<0.001	0.024	0.605	<0.001	<0.001	1	0.635
Sensory Velocity	0.124	<0.001	0.072	0.865	0.006	<0.001	<0.001	1
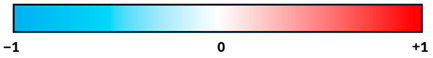								

Cells in the upper triangle display Spearman’s ρ; cells in the lower triangle show two-sided *p*-values. Color legend: red = positive, blue = negative; saturation increases with |ρ| (white ≈ 0). The color scale is fixed from −1 (dark blue) to +1 (dark red) across all correlation tables; diagonal = 1.0 (self-correlation).

## Data Availability

The data presented in this study are available on request from the corresponding author. The data are not publicly available due to privacy and ethical restrictions.
